# Infracommunity crowding as an individual measure of interactive-isolationist degree of parasite communities: disclosing the effects of extrinsic and host factors

**DOI:** 10.1186/s13071-016-1371-2

**Published:** 2016-02-17

**Authors:** Nicola Ferrari, Carlo V. Citterio, Paolo Lanfranchi

**Affiliations:** Department of Veterinary Sciences and Public Health, Università degli Studi di Milano, Via Celoria 10, 20133 Milan, Italy; Istituto Zooprofilattico Sperimentale delle Venezie – SCT2, Belluno, Italy

**Keywords:** Co-infection, Interactivity, Isolationism, Parasite-parasite interaction, Infracommunity structure, Ruminants, Abomasal nematodes

## Abstract

**Background:**

Interactions between parasite species within a host play a fundamental role in shaping parasite communities that have been classified within a continuum between interactive and isolationist. Interactive communities are principally structured by interactions between parasite species, while isolationist communities are structured by processes independent of the presence of other parasite species. Assessing whether, and to what extent, parasite communities exist along this continuum has been challenging due to a lack of an index that quantifies the degree of interactivity. Moreover, the absence of an index at the individual host level has made it unfeasible to identify host and extrinsic factors that may influence the degree of interactivity of a parasite community.

**Methods:**

Here we propose an infracommunity crowding index that can reflect the degree of interactivity of a parasite community within each individual. This index quantifies the mean number of parasites that the average parasite within a community is exposed to, including the different aspects of parasite communities important in determining the level of interactivity, i.e. total abundance, species richness and evenness. We applied this analytical approach to the abomasal parasite communities of three alpine ruminant species that are traditionally viewed as isolationist.

**Results:**

The application of our index to abomasal parasite communities shows that the majority of parasites live in highly crowded communities, suggesting that these host species harbour interactive parasite communities. In addition, the infracommunity crowding was highly variable and influenced by the host species, as well as by the timing of sampling and host age and sex.

**Conclusions:**

Despite increasing evidence on the influence of interactions between parasite species in shaping infections, an analytical measure to quantify the degree of interactivity of parasite communities is lacking. Here we present a new analytical approach which, when applied to parasite communities, appears to be sensitive to both extrinsic and host factors, highlighting that the degree of interactivity is not a static and specific feature of host species, but rather a dynamical process that keeps evolving during host’s life. The new index provides opportunities for further investigations aimed at revealing the determinants of parasite interactivity.

## Background

Animals are frequently infected by multiple parasite species and thus in each individual host a community, referred to as an infracommunity [1, 2], may be established. Parasitologists have investigated the origin and evolution of the structure of these communities and, among the different proposed hypotheses, Holmes & Price [3] focused on the role of interactions between parasite species, formulating the interactive vs. isolationist classification. According to their hypothesis, communities can be assigned into two extreme groups, depending on whether parasite interactions have an evolutionary role and play a structuring role or whether these roles can be considered as negligible. Holmes & Price [3] suggested that the key points to identify a community as interactive include the presence of many parasite species with high infection rates, a high number of co-infections and large infection niche overlap, as these characteristics may promote a high potential for interspecific interactions. Conversely, the key elements proposed for an isolationist parasite community are low numbers of parasite species and low infection rates, leading to small infrapopulations and few co-infections. These features lead to a low potential for interspecific interactions and thus the community structure is shaped by the individual infection rates of each parasite species rather than by their interactions. The classification of parasite communities by Holmes & Price [3], although influential, presents some practical problems for its application. The two extremes of the interactive vs. isolationist communities can be easily identified if all of the features classifying them as either isolationist or interactive are present. However, in natural systems, a continuum between the extremes is likely to exist [4–9]. As such, this classification may be limiting, not allowing for a quantification of the degree of interactivity/isolationism that may occur between parasite communities. Therefore, the assignment of communities to one of these two extreme classes has mostly involved a purely qualitative assessment, or has been achieved through analytical approaches based on a restricted number of the features included in Holmes & Price's definitions [10]. On the one hand, these diversified approaches hinder comparisons between studies and, on the other hand, they make it difficult to quantify the effects of extrinsic and host factors promoting either isolationism or interactivity.

Dove [11], and later Poulin & Luque [12], proposed interactivity indices in order to quantify the degree of interactivity/isolationism of parasite communities. These indices are based on the accumulation curves of the number of species identified in a host sample, giving a single mean value of the interactivity/isolationism for the entire host population. However, parasite infections are known to vary greatly between individuals and several extrinsic and host factors have been identified as determinants of these heterogeneities [13]. In particular, identification of the factors affecting parasite abundance, species richness and community evenness [4–9, 13, 14] is possible due to the fact that all these parameters assume values that are quantifiable at the level of individual host.

In the absence of a measure which can quantify the degree of interactivity/isolationism within each single individual host, analyses of the comparative effect of host and extrinsic factors on parasite community structure are difficult. To address this issue, we developed an index called “infracommunity crowding” by extending the concept of crowding previously proposed by Lloyd [15] which measures the “number of other individuals” experienced by a single individual. More in details, the Lloyd’s measure measures the group size perceived by a group member as opposed to the group size measured from an outsider’s viewpoint (e.g. intensity of infection or population density) and is referred exclusively to intraspecific interactions [16]. Here, we translate Loyd’s concept to interspecific interactions occurring within a community, obtaining an index which represents the number of individuals of other species that the average parasite individual of that community may establish a relationship with. Thus, following statement of the Bush & Lotz [17] that “not all the competitive interactions involve crowding but all crowding events involve competition”, if we condense the crowding that the community experiences into a single number, we can use it as an index of interactivity/isolationism.

The aim of this paper is two-fold. First, we present a new infracommunity crowding index, describe its logical basis, the details of its computation and present some of its properties. We then apply this index to the infracommunities of three mountain ruminant species which, through analyses on a restricted number of different characteristics, had been previously been viewed as isolationist [18–20]. Using our dataset, we finally quantify infracommunity crowding and analyse the influence of host (species, age, sex) and extrinsic factors (year, month) on infracommunity crowding.

## Methods

### The infracommunity crowding index

**Fig. 1:**
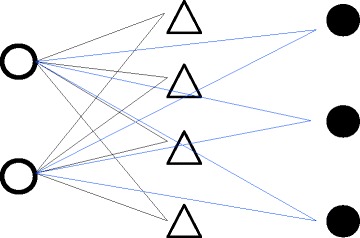
Representation of the hypothetical total number of interactions individuals of species A may have with individuals of species B and C

The infracommunity crowding index (hereafter ICr) is calculated by averaging the crowding each species experiences from other species over the total number of individual parasites within the infracommunity. Essentially, the crowding each species experiences is the number of all possible interactions its individuals may have with all individuals of any other species, excluding conspecifics (Fig. 1).

Thus, if we define *x*_*a*_, *x*_*b*_ and *x*_*c*_ as the number of individuals of species A, B and C, respectively, each individual of species A may establish interspecific interactions with *x*_*b*_ + *x*_*c*_ and the whole crowding of species A can be expressed by *x*_a_*(*x*_*b*_ + *x*_*c*_).

Averaging the crowding of all species over the total parasite abundance we obtain:

1 $$ ICr=\frac{2\ast {\displaystyle \sum_{j=1}^{S-1}\left({x}_j\ast {\displaystyle \sum_{i=j+1}^S{x}_i}\right)}}{N} $$

where *x*_*j*_ represents the abundance of the j-th parasite species, *S* the total number of parasite species (hereafter species richness) within the infracommunity and *N* the total parasite abundance (hereafter total abundance).

To demonstrate how to calculate and how we developed the infracommunity crowding index, we do so for the hypothetical parasite community illustrated in Fig. 1, with a richness (*S*) of 3 species and single species abundances x_j_ respectively of 2, 4 and 3 individuals, leading to a total abundance (*N*) of 9 parasites. As such, each parasite of the species A experiences a crowding by 4 parasites of species B and 3 of species C. Hence, the overall crowding suffered by parasites of species A is 2*(4 + 3) = 14. Similarly, the overall crowding experienced by parasites of species B and C is 4*(2 + 3) = 20 and 3*(4 + 2) = 18, respectively. The total infracommunity crowding can be obtained by summing crowding values for the three species and therefore will be (2*(4 + 3)) + (4*(2 + 3)) + (3*(4 + 2)) which can be simplified in: 2*(2*3 + 2*4 + 3*4) and finally rearranged in 2*(2*(4 + 3) + 4*3).

In order to obtain the infracommunity crowding experienced by an average parasite, the total infracommunity crowding must be averaged over the total parasite abundance, thus producing the ICr formula generalised in Equation 1:

$$ ICr=\frac{2\ast \left[2\ast \left(4+3\right)+4\ast 3\right]}{9} $$

Hence, for this hypothetical infracommunity, ICr results in 5.77 parasites/parasite, meaning that the average parasite interacts with a mean number of 5.77 other parasites.

Despite the calculations, we assume that when the richness is smaller than two parasite species, the infracommunity crowding index has a value of 0 since we are not dealing with a community.

The ICr measures the opportunity for the average parasite individual to interact with parasite individuals of other species. Interspecific interactions increase when infracommunities have either more parasites (i.e. high total abundance) and/or more parasite species (i.e. high species richness). Moreover, interspecific interactions should even increase when parasite individuals are more evenly distributed among species. For example, a community with a richness of four species and a total abundance of 100 parasites will provide more opportunities for interspecific interaction when all four species hold 25 parasites each, compared to the case where one species holds 97 parasites and the other three have just one individual each.

The infracommunity crowding index includes all these factors since its computation is based on the total abundance (*N*), species richness (*S*) and different assemblages of species accounting for single species intensities (*x*_*j*_).

### Data on the abomasal parasite communities of alpine ungulates

The infracommunity crowding index was calculated using data on parasite infracommunities of 261 chamois (*Rupicapra rupicapra*), 126 roe deer (*Capreolus capreolus*) and 58 alpine ibex (*Capra ibex*). Alpine ibex were collected from Graubunden (South Switzerland), chamois and roe deer from hunting districts of the Province of Lecco (north Italy). The data were collected during hunting seasons (i.e. September-December) of 1989–1990 and 2007 in Graubunden and 1998–2005 in the Province of Lecco. Data for chamois come from the database analysed by Citterio et al. [20].

The abomasal parasite fauna of the three host species is composed of 11 species of trichostrongylid parasites which can be considered as a guild of species since they use the host resources in a similar way [1, 21]. Nematodes were identified by morphological criteria according to [22–25]. The following parasite morphotypes were considered to represent a single species: (i) *Teladorsagia circumcincta*/*T. trifurcata*/*T. davtiani* (*T. pinnata*) as *T. circumcincta* complex [26]; (ii) *Marshallagia marshalli*/*M. occidentalis* as *M. marshalli* complex [27]; (iii) *Spiculopteragia spiculoptera*/*Rinadia mathevossiani* as *S. spiculoptera* complex [28]; (iv) *Ostertagia leptospicularis*/*O. kolchida* as *O. leptospicularis* complex [29]; and (v) *Osteragia lyrata*/*O. ostertagi* as *O. ostertagi* complex [30].

For each host individual we recorded species, sex, age, month and year of sampling. For each infracommunity we recorded parasite abundance, species richness and evenness (according to [1]). Since the morphological criteria apply only to male nematodes, the abundance of each parasite species has been calculated by doubling the number of the male nematodes collected, assuming a 1:1 sex ratio [20]. The total abundance of parasites within the parasite community was calculated as the sum of all nematodes from all species. Species richness corresponds to the number of species recovered in each host individual. Evenness has been calculated using the Brillouin’s index, as this represents the most appropriate measure for fully censused communities [31]. The Brillouin’s index ranges from 1, when all species are equally abundant, to 0 when a single species dominates the community. These indices for each parasite species are summarised in Table 1.

**Table 1:** Parasite species composition of the abomasal parasite communities in the three host species. Species prevalences and abundances are based on the number of male specimens numbers only

	Chamois (*n* = 261)		Roe deer (*n* = 126)		Alpine Ibex (*n* = 58)	
	Prevalence (95 % CI)	Abundance ± SE	Prevalence (95 % CI)	Abundance ± SE	Prevalence (95 % CI)	Abundance ± SE
*Teladorsagia circumcincta* complex	48.3 (42.2–54.3)	23.3 ± 3.95	20.6 (15.7–25.5)	5.6 ± 2.49	94.8 (92.1–97.5)	463.8 ± 60.59
*Marshallagia marshalli* complex			0.8 (0.0–1.9)	0.1 ± 0.00	94.8 (92.1–97.5)	265.4 ± 38.32
*Spiculopteragia spiculoptera* complex	18.4 (13.7–23.1)	4.1 ± 0.96	93.6 (90.7–96.6)	211.0 ± 21.14		
*Ostertagia leptospicularis* complex	11.5 (7.6–15.4)	2.3 ± 0.89	78.6 (73.6–83.6)	67.5 ± 9.10		
*Ostertagia ostertagi* complex	0.8 (0.0–1.8)	0.2 ± 1.31	0.8 (0.0–1.9)	0.2 ± 0.00	29.3 (23.7–34.8)	11.5 ± 2.30
*Haemonchus contortus*	66.3 (60.5–72.0)	37.0 ± 4.20	17.5 (12.9–22.1)	10.9 ± 8.39	1.7 (0.1–3.3)	1.0 ± 0.00
*Trichostrongylus axei*	25.3 (20.0–30.6)	17.8 ± 12.46	26.9 (21.6–32.3)	26.4 ± 18.00	36.2 (30.4–42.0)	29.8 ± 17.55
*Trichostrongylus capricola*	1.5 (0.1–3.0)	0.1 ± 0.00			10.3 (6.6–14.0)	2.1 ± 2.09
*Trichostrongylus vitrinus*	7.7 (4.4–10.9)	1.3 ± 0.75	3.2 (1.0–5.3)	0.5 ± 0.89	22.4 (17.3–27.4)	11.3 ± 5.59
*Trichostrongylus colubriformis*	3.1 (1.0–5.1)	0.4 ± 0.32	0.8 (0.0–1.9)	0.1 ± 0.00		
*Trichostrongylus longispicularis*	0.4 (0.0–1.1)	0.1 ± 0.00	1.6 (0.1–3.1)	0.5 ± 2.52		
Infracommunities						
Mean richness	1.8 ± 0.08		2.4 ± 0.08		2.8 ± 0.12	
Mean total nematode count	100 ± 0.00	214.2 ± 23.00	100 ± 0.00	832.8 ± 86.89	100 ± 0.00	1875.3 ± 164.44
Mean evenness (Brillouin’s index)	0.4 ± 0.02		0.6 ± 0.03		0.6 ± 0.03	
Component community richness	10		10		7	
Infracommunity crowding	60.3 ± 6.3		266.8 ± 36.0		611.0 ± 61.8	

### Statistical analyses

For each host individual, infracommunity crowding index was calculated using Equation 1.

To investigate ecological sources of community variability, we fitted generalised linear models to explore the effect of host species, sex, age (continuous) and the extrinsic factors month and year of sampling (both considered as discrete) on the following dependent variables: infracommunity crowding (ICr), total abundance, species richness and evenness. Models initially included all first order interactions between the explanatory variables. Terms not significantly contributing to explain the observed variability of the response variable were removed in a stepwise manner, using a likelihood ratio test until we obtained the minimal adequate model [32].

The error distributions producing the best model fits were the Poisson distribution for species richness and negative binomial distribution for ICr, total abundance and evenness.

For the minimal adequate model on infracommunity crowding, we present estimates of the effects of all explanatory variables. Analyses on total abundance, species richness and evenness were mainly run to identify the main factors affecting these parameters, in order to subsequently compare whether host and extrinsic factors had an analogous influence on crowding or not. Therefore, the composition of these minimal adequate models is presented omitting details on the effects of these factors. All analyses were undertaken in R 3.2.2 [33], using MASS package for models with negative binomial distributions. Descriptive statistics of the dependent variables are presented as the mean ± standard error of the mean, whereas for prevalence 95 % confidence intervals are used.

## Results

### Composition and structure of the nematode communities

The abomasal parasite communities of the three host species examined comprised 11 nematode species, all belonging to the nematode family Trichostrongylidae. The total numbers of parasite species detected in each host species were similar, ranging from seven in the alpine ibex to ten species in the chamois and roe deer. Conversely, the infracommunities were more diverse, showing variability both between and within host species (Table 1).

The infracommunity structure was strongly affected by host species (Table 2). In particular, richness was significantly higher in communities in the alpine ibex and roe deer, which harboured 2.8 ± 0.12 and 2.4 ± 0.08 species/individual host, respectively, and lower in those in the chamois with 1.8 ± 0.08 species/individual host (Table 1). Mean abundance was higher in communities in the alpine ibex with 1875.3 ± 164.4 parasites/individual host, whereas those in the roe deer and chamois harboured 867.2 ± 86.8 and 214.2 ± 23.0 parasites/individual host, respectively (Table 1). The evenness was higher in communities in the alpine ibex and roe deer, (0.64 ± 0.03 and 0.62 ± 0.03 parasites/individual host, respectively) than in communities in the chamois (0.43 ± 0.02) (Table 1). The effect of host species on total abundance varied with sampling year and host age (Table 2), whereas its effect on evenness varied with host sex and sampling month (Table 2). Host sex influenced directly only richness and evenness whereas total abundance was affected through interactions with sampling year (Table 2). Host age did not affect directly any of these parameters (Table 2). Finally, temporal variability played a great role with direct effects of month and sampling year. In particular, richness, total abundance and evenness differed between years and the latter two parameters varied even with sampling months. Additionally, the monthly variability of evenness differed between sampling years and with host age (Table 2).

**Table 2:** Factors affecting species richness, total abundance and infracommunity evenness

Factor	Richness	Abundance	Evenness
Species	<0.001	<0.001	<0.001
Sex	<0.001	0.051	0.011
Age	0.970	0.703	0.244
Month	0.117	<0.001	0.001
Year	0.002	<0.001	0.004
Species : Age		0.003	0.015
Species : Year		0.012	
Species : Sex			0.002
Species : Month			<0.001
Sex : Year		0.043	
Sex : Month			0.011
Month : Age			0.026
Month : Year			0.009

### Patterns of infracommunity crowding

The infracommunity crowding index in nematode communities of the three host species studied showed an aggregated distribution with a significant fit to the negative binomial distribution (Deviance = 1329, df = 5779, *p* = 1, maximum likelihood estimate of k = 0.23). This distribution implies that most parasites live in crowded communities: 67 % of all parasite individuals sampled were indeed recovered in the hosts harbouring the top 20 % most crowded infracommunities.

**Fig. 2:**
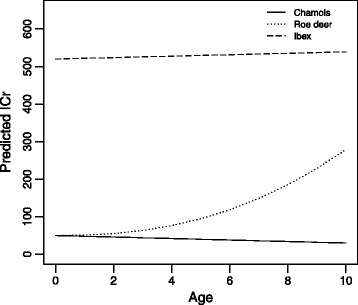
Model predicted effect of host age on the infracommunity crowding in the chamois, roe deer and alpine ibex abomasal helminth communities. The other explanatory variables were held as: sex = male; year = 2000; month = November

Among the factors affecting the infracommunity crowding, host species was highly influential with the ibex harbouring the most crowded communities with 611.0 ± 61.8 parasites/parasite and the chamois harbouring the least crowded with 60.3 ± 6.3 parasites/parasite. Communities in the roe deer showed an intermediate value of 266.8 ± 36.0 parasites/parasite (Table 3). Host age had a different effect depending on the host species: communities tended to be less crowded with increasing age in the chamois whereas in the ibex there was a slight crowding increase with age and in the roe deer infracommunity crowding sharply increased with age (Fig. 2). Finally, the infracommunity crowding was influenced by the timing of sampling with direct and indirect effects of month and year. In particular, host species, sex and month effects showed different responses in different years.

**Table 3:** Factors affecting community crowding (ICr)

Factor	Parameter estimate ± SE	df	Deviance	P value
Species		2	270.16	<0.001
Chamois	0.00			
Roe deer	−1.15 ± 0.58			
Ibex	0.97 ± 0.79			
Sex		1	1.96	0.161
F	0			
M	0.44 ± 0.26			
Age	−0.04 ± 0.02	1	0.14	0.700
Month		4	23.84	<0.001
Year		9	23.50	0.005
Species: Year		4	35.14	<0.001
Species: Age		2	11.16	0.003
Sex: Year		8	16.53	0.035
Month: Year		22	51.97	<0.001

## Discussion

We propose infracommunity crowding index (ICr) as a measure to quantify the degree of isolationism/interactivity of parasite infracommunities. This measure expresses the number of parasite individuals of other species that an average individual of a parasite community can establish an interaction with. Moreover, since the index accounts for parameters which reflect other aspects of the isolationism/interactivity degree (i.e. total parasite abundance, number of species and their evenness), it also provides a measure to quantify this continuum.

The application of this index to a dataset of abomasal parasite communities characterised by high variability of total abundance, species richness and evenness, revealed a high variability in infracommunity crowding. This variability was principally due to host species, timing of sampling but also, to a lesser extent, to host age and sex. These results emphasise that the interactive nature of a community should not be viewed as a static characteristic but rather as a dynamic feature evolving and shifting through time and between host individuals.

Studies about interactions among parasite species have fostered our understanding of the role such interactions may play in influencing parasite infections and shaping communities [34–38]. However, the lack of adequate measures to quantify parasite interactions at the community level has hampered these investigations so that most recent studies approached these topics through pairwise analyses between parasite species [35–38]. Although pairwise analyses can provide robust results [39], this analytical approach shifts the attention from the community level to a population ecology point of view [40] where each set of interactions between pairs of species is analysed singularly. As a consequence, this approach may lead to potentially overlook the emerging properties rising from multiple interactions which characterise communities [40]. Infracommunity crowding index can provide this measure at the community level.

However, a limitation of community level measures resides in their constraint to reveal presently occurring mechanisms: for example, in the present study the infracommunity crowding observed may be a result of previous events without informing on the ongoing interactions. In these instances, null models can predict results from hypothesised conditions, thus giving baseline values for comparison with observed results [41]. The development of this approach for infracommunity crowding would supply expected values for the opposite condition of interactivity or isolationism, allowing a comparison of the observed values. Besides the development of null models, another future step to improve the interpretation of infracommunity crowding will be a deep sensitivity analysis, aimed at elucidating the relative contribution of abundance, richness and evenness on crowding. Preliminary analyses on simulated data, showed that infracommunity crowding increases with increasing values of abundance, richness and evenness; however, more comprehensive analyses would allow to disclose the relative contribution of these epidemiological characteristics and their synergistic effects.

Compared to previous measures based on accumulation curves [11, 12], infracommunity crowding index allows an extensive analysis of the effects of extrinsic and host factors, since it is computed for each individual host. Moreover, since infracommunity crowding represents the number of individuals of other species that an average individual within a community experiences, it represents an absolute measure thus allowing for direct and biologically meaningful comparisons between species, samples, and sites from different studies.

Parasite interactions may be based on different mechanisms, such as direct interference, competition for resources or host-mediated processes, such as those mediated by the immune system [36, 42–44]. Interactions may thus be established between parasites sharing the same organs and anatomical systems [35], but also between parasites living in different locations (e.g. stomach and skin; see [45, 46]) and even between parasite species with very distant taxonomic relationships (e.g. helminths and ticks or protozoa and viruses [46–48]). Even if infracommunity crowding index can be visualised more intuitively as a direct contact between parasites, its computation is not based on the biological mechanisms of interaction between parasites. Therefore, this index can be easily computed for several forms of parasite community, from those limited to a specific organ to those including the entire host organism and composed of any taxonomic mix of parasites. It must be noted that, in its present form, infracommunity crowding index presents the limit of being calculated based on parasite abundances (no. of parasites/host individual), thus excluding those parasites where counting is not feasible or meaningful, such as microparasites, haemoprotozoans or cestodes. In particular, regarding cestodes, parasite burden is better evaluated through parasite biomass rather than abundance [49, 50]. In this case, calculation of infracommunity crowding index would not be biologically sound, but it would appear feasible to calculate the index by scaling parasite abundances to their biomasses through an appropriate correction coefficient.

The application of infracommunity crowding index to abomasal parasite communities in the alpine ungulates studied revealed an aggregated distribution with a small proportion of hosts harbouring the most crowded parasite communities. This implies that the vast majority of parasites live in highly crowded communities and can establish interactions with other species, suggesting that, in abomasal parasite communities of ruminants, parasite interactions may have a prominent evolutionary and structuring role and that these communities can be viewed as interactive. This interpretation contrasts with previous studies that classified parasite communities of alpine ruminants as being “typically” isolationist [18–20]. In particular, conclusions by [20] were drawn on a subsample of animals (i.e. chamois) that is actually included in the present dataset, but that had been previously analysed using a limited number of community parameters and in a host health management rather than a parasite community ecology perspective. This indicates that infracommunity crowding, by including a greater number of community features, can provide a more exhaustive picture and take into account the variability along the continuum between the two extremes, isolationism and interactivity.

The abomasal parasite infracommunities of the three alpine ruminants were characterised by a high variability of parasite species richness, total abundance and evenness. However, while a wide set of host and extrinsic factors has been identified as influencing these three parameters, the infracommunity crowding index was found to be affected mainly by host species and by the timing of sampling, with a less pronounced effect of host age and sex. Thus, although each host species seems to hold distinctive parasite communities with respect to their degree of crowding, the effect of time and host factors indicates that interactivity is not a fixed host species characteristic [3, 18, 20], but it should be viewed as a variable and dynamical process evolving throughout the host’s life. This latter result thus implies that the degree of interactivity should not be viewed as a host species-specific feature and, at the same time, it leads to reconsider the evolutionary role of interactivity in structuring parasite communities [3], depending on its temporal occurrence and variability between host individuals.

## Conclusions

In the present study we propose the use of infracommunity crowding index (ICr) as a new measure to evaluate the degree of isolationism/interactivity of parasite communities. This measure takes simultaneously into account different features of parasite communities, i.e. total abundance, species richness and evenness, known to be important in determining the level of isolationism/interactivity. As a further step, we need to elucidate the relative contribution of these features to infracommunity crowding and formulate null models against which to test isolationist or interactivity conditions. The application of infracommunity crowding index to a field dataset of parasite communities in alpine ruminants suggests, contrasting to previous results, that such communities exhibit high levels of interactivity. Thus their former isolationist classification, although functional for analyses on health impact of parasites, should be revised. Moreover, the influence of host species, age and sex and sampling time suggests that interactivity is a dynamic process that evolves during host’s life rather than host species-specific feature. In this sense, rather than simply investigating whether a community is interactive or isolationist, new questions may regard the “degree of interactivity” of communities, which can be scaled along a continuum. Moreover the infracommunity crowding index offers an absolute measure that can be compared to indices of other communities. This study is a step forward in our investigations on the role of parasite interactions as forces structuring infracommunities since it provides a new analytical approach which opens to a broader overview on the extent of interactivity and on the factors promoting it.
